# Importance of coverage and quality for impact of nutrition interventions delivered through an existing health programme in Bangladesh

**DOI:** 10.1111/mcn.12613

**Published:** 2018-04-14

**Authors:** Phuong Hong Nguyen, Edward A. Frongillo, Tina Sanghvi, Sunny S. Kim, Silvia Alayon, Lan Mai Tran, Zeba Mahmud, Bachera Aktar, Purnima Menon

**Affiliations:** ^1^ Poverty, Health and Nutrition Division International Food Policy Research Institute Washington DC USA; ^2^ University of South Carolina Columbia SC USA; ^3^ FHI360 Washington DC USA; ^4^ BRAC James P Grant School of Public Health BRAC University Dhaka Bangladesh

**Keywords:** Bangladesh, implementation, maternal nutrition, service delivery

## Abstract

Understanding implementation of interventions is critical to illuminate if, how, and why the interventions achieve impact. Alive & Thrive integrated a nutrition intervention into an existing maternal, neonatal, and child health (MNCH) programme in Bangladesh, documenting improvements in women's micronutrient supplement intake and dietary diversity. Here, we examined how well the nutrition intervention was implemented and which elements of implementation explained intervention impact. Survey data were collected in 2015 and 2016 from frontline health workers (FLW) and households in areas randomized to *nutrition‐focused MNCH* (intensified interpersonal counselling, community mobilization, distribution of free micronutrient supplements, and weight‐gain monitoring) or *standard MNCH* (antenatal care with standard nutrition counselling). Seven intervention elements were measured: time commitment, training quality, knowledge, coverage, counselling quality, supervision, and incentives. Multiple regression was used to derive difference‐in‐differences (DID) estimates. Using village‐level endline data, path analysis was used to determine which elements most explained intervention impacts. FLWs in both areas were highly committed and well supervised. Coverage was high (>90%) for counselling, supplement provision, and weight‐gain monitoring. Improvements were significantly greater for nutrition‐focused MNCH, versus standard MNCH, for training quality (DID: 2.42 points of 10), knowledge (DID: 1.20 points), delivery coverage (DID: 4.16 points), and counselling quality (DID: 1.60 points). Impact was substantially explained by coverage and delivery quality. In conclusion, integration nutrition intervention into the MNCH programme was feasible and well‐implemented. Although differences in coverage and counselling quality most explained impacts, all intervention elements—particularly FLW training and performance—were likely important to achieving impact.

**Key messages**

- Integration of the maternal nutrition intervention (including intensified interpersonal counselling, community mobilization, distribution of free micronutrient supplements, and weight‐gain monitoring) into the maternal, neonatal, and child health programme in Bangladesh was feasible and well‐implemented.
- Among seven elements of intervention (time commitment, training quality, knowledge, coverage, counselling quality, supervision, and incentives), differences in coverage and counselling quality most explained impacts, although all intervention elements—particularly FLW training and performance—were likely important to achieving impact.
- This paper provides insights on where and how maternal nutrition interventions can be integrated with and delivered through routine healthcare services.

## 1 INTRODUCTION

Comprehensive programme evaluations should examine not only the impacts produced by an intervention but also the mechanisms and elements of interventions that influence their impacts (Campbell et al., 2000; Damschroder et al., 2009; Fixsen et al., 2005). Understanding the implementation processes of interventions is critical to illuminate if, how, and why the interventions achieve impact. In addition, implementation data are needed to test theories driving the assumptions of the intervention and to accurately interpret findings. Timely use of implementation data can also support informed decision‐making for programme adjustments and continuous quality improvements (Duerden & Witt, 2012).

Implementation of interventions consists of several components, among them how interventions are implemented as planned (fidelity or adherence to design), the amount that has been delivered (dosage or quantity), how well different intervention components have been conducted (quality), participation rates (reach), and participant responsiveness (Carroll et al., 2007; Saunders, Evans, & Joshi, 2005). There is strong evidence that effective implementation is associated with better impacts of intervention. A review of more than 500 studies of health programmes reported that high level of implementation, including those components named above, was associated with at least two or three times higher impact in promotion (mental and physical health) as well as prevention programmes (alcohol, tobacco, and substance abuse; Durlak & Dupre, 2008). The review further highlighted the lack of or limited implementation of data in most studies and that the assessment of implementation is a necessity in programme evaluations; implementation data are needed to document what was conducted and how outcome data should be interpreted (Durlak & Dupre, 2008).

There is increasing evidence of the critical role of implementation science in the field of nutrition, particularly on maternal and child health programmes (Leroy & Menon, 2008; Loechl et al., 2009; Menon et al., 2008; Robert et al., 2006; Robert et al., 2007) and on promotion of infant and young child feeding practices (Avula et al., 2013; Kim et al., 2015; Nguyen et al., 2014; Rawat et al., 2013). Despite growing literature, research that links implementation elements and nutrition impact is limited, and more attention to process‐oriented research is needed that can shed light on how nutrition interventions can be operationalized effectively to achieve desired outcomes (Leroy & Menon, 2008; Shekar, 2008; Stoltzfus, 2008).

Maternal undernutrition is a significant public health problem in Bangladesh; more than half of all pregnant women are anaemic (Hyder, Persson, Chowdhury, Lonnerdal, & Ekstrom, 2004), and nearly a quarter of the women of reproductive age are undernourished or underweight (BMI < 18.5 kg/m^2^; National Institute of Population Research and Training, 2011), mainly due to poor diets and low intake of micronutrient supplements (Arsenault et al., 2013), among other determinants. To address these challenges, the Alive & Thrive initiative, in collaboration with a large national non‐governmental organization in Bangladesh (BRAC), integrated a maternal nutrition‐focused, multi‐component behaviour change intervention into its existing maternal, neonatal, and child health (MNCH) platform. We have previously shown that the intervention successfully improved multiple outcomes such as maternal dietary diversity, micronutrient supplement consumption, and exclusive breastfeeding practice (Nguyen et al., 2017). For this paper, our objectives were to (i) examine how well the integrated nutrition interventions were implemented and (ii) identify which elements of implementation were most important in explaining intervention impacts on three outcomes: consumption of iron and folic acid (IFA) and calcium supplements, and dietary diversity.

## 2 METHODS

### 2.1 Study context and intervention description

This study is part of an evaluation on the feasibility and impact of integrating intensified maternal nutrition interventions into the existing MNCH programme in Bangladesh. Detailed description of the intervention, the study design, data collection, and main results has been reported elsewhere (Nguyen et al., 2017). Briefly, Alive & Thrive designed a *nutrition‐focused MNCH* interventions (including intensified interpersonal counselling, distribution of free IFA and calcium supplements, weight‐gain monitoring, community mobilization, and family engagement), targeted to pregnant and recently delivered women, with the overall goal of improving maternal nutrition. In the *standard MNCH* areas, women received antenatal care with standard nutrition counselling, which had fewer number of visits and much less nutrition content or emphasis.

BRAC delivered the nutrition‐focused MNCH intervention through two types of existing frontline workers (FLWs)—*Shasthya Kormi* (community health workers) and *Shasthya Shebika* (community health volunteers). As part of routine antenatal care, health workers conducted monthly home visits for all pregnant and recently delivered women to (i) demonstrate and counsel on a specific diet plan (both quality and quantity of food groups), (ii) provide free supplements (IFA and calcium tablets) and advice on using them, (iii) measure weight and explain optimal weight‐gain patterns, (iv) counsel on adequate rest during pregnancy, (v) promote optimal breastfeeding practices, and (vi) engage husbands and other family members to ensure enough varied foods and supplements are available and to support pregnant women to consume them. In total, health workers were tasked with conducting seven visits during pregnancy and five visits during postpartum (within 6 weeks of delivery). Health volunteers were asked to conduct two visits per household per month and provided follow‐up messages to the women and family members to reinforce the demonstrations and counselling given by health workers.

To assist with counselling, a sample diet chart was developed through formative research, tested in the field, and then revised. The diet chart described recommended quantities of diversified food for each trimester with examples of foods from five food groups. A nutrition calendar was developed including the diet chart, a monthly weight‐tracking chart, and key messages. Health workers were trained on using the nutrition calendar while counselling pregnant women and delivered mothers about dietary diversity and recommended quantity of food; counselling included finding diversified food from locally available low‐cost food.

BRAC integrated nutrition‐focused monitoring, supervision, and support into their routine MNCH services. The programme staff regularly monitored the maternal nutrition‐related activities through a checklist to identify the following: (i) gaps in the coverage and quality of service delivery so that appropriate actions could be taken to improve FLW performance in a timely manner; and (ii) high‐performing FLWs so that they can be recognized and rewarded for their work. The performance improvement cycle involved household listing ➔ allocation of work areas ➔ hands‐on training of FLWs ➔ monthly meeting/refresher ➔ service delivery support through supplies and job aids ➔ supportive supervision and routine monitoring feedback ➔ performance‐based cash incentives ➔ continued refresher training. In the nutrition‐focused MNCH areas, health volunteers received performance‐based cash incentives based on home visits conducted and mothers practicing the recommended behaviours. The pre‐existing MNCH programme's schedule of cash incentives was continued in both areas, including the standard MNCH areas, and included reaching a newly identified pregnant woman and if a mother practiced early initiation of breastfeeding.

### 2.2 Study design and participants

**Figure 1:**
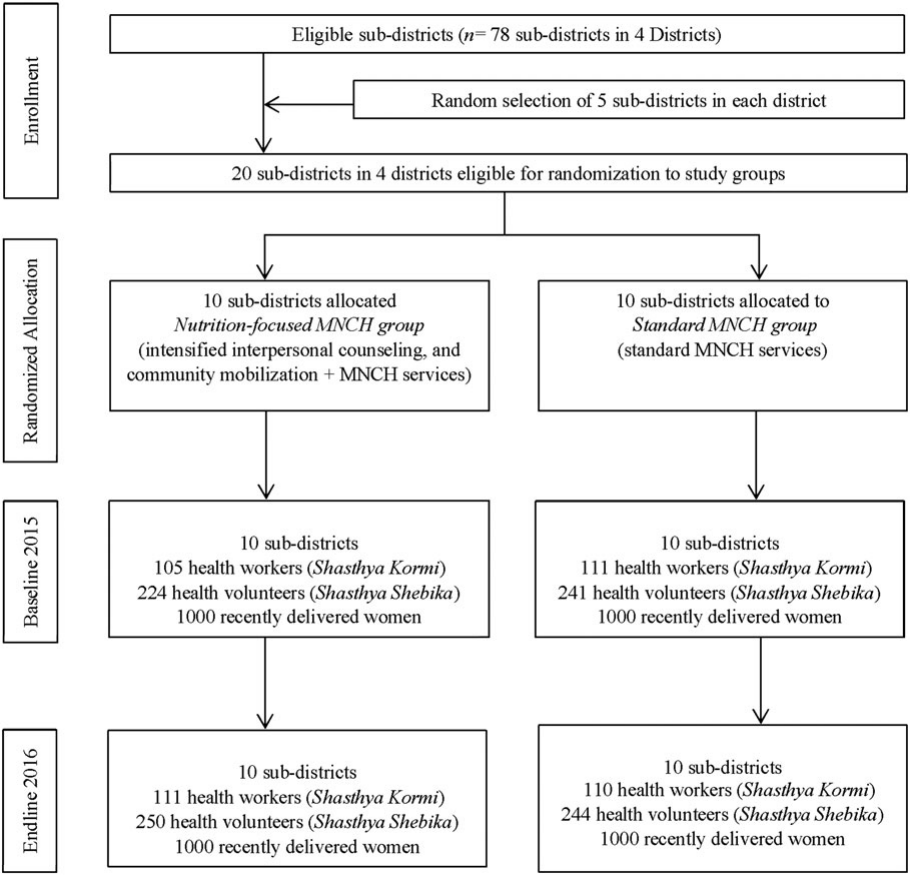
Trial profile

This study used a cluster‐randomized impact design in which 20 rural *upazilas* (subdistricts) were randomly assigned to either nutrition‐focused MNCH or standard MNCH interventions. Details of the study design and sample size calculation have been described in detail elsewhere (Nguyen et al., 2017). Survey data from FLWs and households were collected through two cross‐sectional surveys in 2015 and after 1 year of implementation in 2016 (between June and August at both time periods). In total, 1,000 recently delivered women with children <6 months of age, 110 health workers, and ~250 health volunteers from 85 villages were surveyed in each group and each survey round (Figure 1).

This study received ethical approval from the Bangladesh Medical Research Council and the Institutional Review Board at the International Food Policy Research Institute. All participants were provided with detailed information about the study both in writing and verbally, and informed consent was obtained prior to participation.

### 2.3 Measurements

The consumption of IFA and calcium supplements was assessed among recently delivery women by asking women to report how many IFA or calcium tablets they consumed during their last pregnancy. During monthly visits to women's homes, FLWs recorded the number of IFA and calcium tablets consumed in a mother–baby book as part of the MNCH programme; this book was used to assist women in their recall. Maternal dietary diversity was assessed using a 24‐hr dietary recall; women were asked to describe all foods and beverages they consumed the preceding 24 hr; these food items were then grouped into 10 food groups categories following the Women Dietary Diversity Score classification (Fao & Fhi360, 2016). The dietary diversity measure is the number of food groups consumed and thus ranged from 0 to 10.

Intervention implementation was measured using seven elements: FLW's time commitment, quality of training, knowledge, coverage of service delivery, quality of counselling service, supervision, and incentives. FLW's time commitment was measured by a set of items related to the numbers of days worked and conducted home visits in a month, the time they spent for each home visit and discussed maternal and child nutrition, and the number of pregnant women and children under 6 months of age in their catchment areas.

The training indicator was constructed based on items related to FLW's participation in full training and refresher training, time from last refresher training, and topics discussed at the last full training. Total quality of training score was created by adding scores from these items.

FLW's knowledge on maternal nutrition was assessed from responses to items about the benefits of adequate nutrition, types of food that pregnant women should eat every day, duration and benefit of taking IFA or calcium supplements during pregnancy, and knowledge of the foods that interfere with IFA absorption. Each knowledge item was given a score of 1 (correct) or 0 (incorrect), and the sum was used as the knowledge score.

Coverage of service delivery was measured by the percentage of women who received home visits from FLWs, the number of home visits during pregnancy (score 1 if >4 visits), whether women were provided with IFA and calcium supplements or with any dietary advice, and whether women had weight measured. Each service that women received was given a score of 1, and the sum was used as the coverage of service delivery score.

Quality of counselling service was assessed by the percentage of women who received various messages on pregnancy care including dietary quantity and quality, taking IFA and calcium tablets, gaining weight, taking rest, and avoiding heavy workload. Each message women received was given a score of 1, and the sum was used as the quality of counselling service score.

FLW's perceptions about supervision were measured by a set of 15 items related to supportive feedback and exchange, and help to manage stress and workload. FLWs were asked to indicate their degree of agreement with each statement, using responses on a 5‐point Likert scale. A summed scale was constructed for the supervision score.

Only heath volunteers received incentives from BRAC. Incentives were assessed by asking health volunteers whether they ever received incentives from BRAC, if they received incentives in the last month, and for which services they received incentives.

The total scores for each element of implementation were rescaled to the theoretical range of 0 to 10 for comparability across elements. Other FLW characteristics that may influence FLW's performance were examined, including age, duration of time worked with BRAC, and education level.

### 2.4 Data analysis

Descriptive analyses were used to report the characteristics of the study samples. Means and standard deviations were calculated for continuous variables, and proportions were calculated for categorical variables. To address the first study objective, we derived difference‐in‐difference (DID) estimates for each element of intervention implementation (except incentives) using regression models that estimated the difference in changes over time between the two study groups (Gertler, Martinez, Premand, Rawlings, & Vermeersch, 2011). All models accounted for clustering at the district and subdistrict level using a cluster sandwich estimator (Hayes & Moulton, 2009).

To address the second study objective, we aggregated endline FLW and household data to the village level. To evaluate the assumption for path analysis of no interaction between exposure and mediator (Vanderweele, 2015), we tested if there was an interaction between study group and each implementation element separately. For each implementation element where there was an interaction, we report the results of the regression models with interaction. For each implementation element where there was no interaction, we used a path model (Kline, 2011) to examine how much of the programme differences in intake of IFA supplements, calcium supplements, and dietary diversity was explained through that element; the indirect effect for each outcome indicator was calculated as the product of the unstandardized regression coefficients for the paths from intervention group to element and from element to outcome.

Data analysis was performed using Stata 14. Results for health workers and health volunteers were similar, so we present results for health workers only (with the exception of the incentives element, which is based on data from health volunteers).

## 3 RESULTS

### 3.1 Sample characteristics

On average, health workers were 30 years old with 5–6 years of working with BRAC; almost all had high school education or higher (results not shown). No differences were observed on the size of target populations in the catchment areas of FLWs. The characteristics of FLWs were balanced between nutrition‐focused MNCH and standard MNCH areas at baseline, indicating that randomization was successful.

### 3.2 Impact on elements of implementation

Overall, improvements were significantly greater in the nutrition‐focused MNCH areas, compared with those in the standard MNCH areas, for total quality of training score (DID: 2.4 points of 10), FLW's knowledge (DID: 1.2 points), delivery coverage (DID: 4.2 points), and counselling quality (DID: 1.6 points; Table 1). Time commitment and perceptions about supervision were high in both groups at both baseline and endline (score > 8 out of 10), with no observed differences between groups. Incentives received were higher among health volunteers in the nutrition‐focused MNCH areas compared with those in the standard MNCH areas (35% vs. 10%).

**Table 1:** Impact of interventions on elements of implementation

	Baseline		Endline		DID^a^ (95% CI)
	Nutrition‐focused MNCH	Standard MNCH	Nutrition‐focused MNCH	Standard MNCH	
	*n* = 105	*n* = 111	*n* = 111	*n* = 110	
	Mean ± SD (%)	Mean ± SD (%)	Mean ± SD (%)	Mean ± SD (%)	
Time commitment					
No. of days of the month usually worked as a health worker	24.9 ± 1.7	25.4 ± 1.6	24.8 ± 1.9	25.0 ± 1.3	0.44 (−0.96, 1.83)
No. of days of the month usually made home visits	18.4 ± 5.7	18.3 ± 5.6	20.1 ± 4.5	19.8 ± 3.5	0.14 (−3.04, 3.33)
No. of home visits usually made each day	19.8 ± 4.2	19.4 ± 4.2	20.5 ± 3.4	20.5 ± 3.6	−0.22 (−2.20, 1.76)
Time usually spent in each home visited (minutes)	16.4 ± 7.4	14.9 ± 6.3	16.2 ± 6.3	16.9 ± 7.5	−3.21 (−6.41, −0.01)
Time usually spent discussing maternal and child nutrition during home visits (minutes)	10.6 ± 7.3	11.2 ± 7.8	15.8 ± 8.9	13.4 ± 7.4	2.94 (0.06, 5.94)
Quality of training score (1–10)	2.5 ± 2.0	2.6 ± 1.7	4.7 ± 2.1	2.4 ± 1.6	2.42 (1.34, 3.50)
Knowledge score (1–10)	6.5 ± 1.3	6.0 ± 1.2	7.5 ± 1.1	5.9 ± 1.2	1.21 (0.42, 2.00)
Coverage of service delivery score (1–10)	5.5 ± 2.3	5.8 ± 2.5	9.4 ± 1.6	5.5 ± 2.5	4.2 (3.28, 5.05)
Quality of counselling service score (1–10)	3.8 ± 1.3	3.8 ± 1.4	5.6 ± 1.4	3.9 ± 1.3	1.6 (0.70, 2.49)
Supervision score (1–10)	8.9 ± 0.7	8.4 ± 0.8	8.4 ± 0.7	8.4 ± 0.8	−0.28 (−0.94, 0.38)
Incentive,^b^ %	–	–	34.8	9.8	25.0 (17.9, 32.0)

*Note*. CI = confidence interval; DID = difference‐in‐difference; MNCH = maternal, neonatal, and child health.

a Difference‐in‐difference impact estimate between baseline and endline adjusted for clustering effect at district and subdistrict levels.

b Incentive for health volunteer.

### 3.3 Time commitment

Health workers worked full‐time for BRAC (25 days per month) and conducted home visits nearly every day (Table 1). Compared with health workers in the standard MNCH areas, those in the nutrition‐focused MNCH areas spent more time discussing maternal and child nutrition in each home visit.

### 3.4 Exposure to and quality of training

The proportions of health workers that received basic training was uniformly high across groups and exceeded 80% (Table 2). At endline, health workers in nutrition‐focused MNCH areas attended monthly refresher trainings on a more regular basis than their counterparts in standard MNCH areas. Training content was also more comprehensive in nutrition‐focused MNCH than that in standard MNCH areas, with more topics on counselling approach (61% vs. 45%), preparing diet chart and calculating food intake (70% vs. 13%), measuring and recording weight (69% vs. 8%), counting and recording of IFA and calcium tablet consumption (68% vs. 12%), and engaging husbands and family members (25% vs. 8%).

**Table 2:** Quality of training received by health workers, by study group and survey round

	Baseline		Endline		DID^a^ (95% CI)
	Nutrition‐focused MNCH	Standard MNCH	Nutrition‐focused MNCH	Standard MNCH	
	*n* = 105	*n* = 111	*n* = 111	*n* = 110	
	Mean ± SD (%)	Mean ± SD (%)	Mean ± SD (%)	Mean ± SD (%)	
Ever received basic training on maternal nutrition	79.8	87.4	97.3	91.8	13.0 (−8.47, 34.5)
Attended monthly refresher training	66.7	80.6	100.0	100.0	14.1 (−16.5, 44.6)
Time from last refresher training received (months)	0.47 ± 0.37	0.31 ± 0.32	0.6 ± 0.6	1.2 ± 3.0	−0.71 (−1.28, −0.14)
Topics discussed at the last full training					
Current situation of maternal health/nutrition and breastfeeding	41.7	40.2	49.1	51.5	−4.57 (−34.6, 25.5)
Importance of maternal nutrition and breastfeeding	34.5	47.4	44.4	47.5	9.37 (−19.5, 38.3)
Counselling approach	51.2	55.7	61.1	44.6	20.6 (−13.5, 54.6)
Preparing diet chart and calculating food budget	32.1	25.8	70.4	12.9	51.7 (15.9, 87.5)
How to measure and record weight of the pregnant women	25.0	20.6	68.5	7.9	56.0 (29.8, 82.3)
Counting and recording of IFA and calcium tablet consumption	39.3	29.9	67.6	11.9	46.4 (27.7, 65.2)
How to engage husbands and other family members	16.7	8.3	25.0	7.9	8.65 (−13.0, 30.3)
Technique of counselling breastfeeding issue	33.3	35.1	40.7	35.6	6.25 (−21.9, 34.4)
How to express breast milk	16.7	19.6	31.5	23.8	10.3 (−15.0, 35.6)
Early initiation of breastfeeding	31.0	35.1	44.4	38.6	9.49 (−14.2, 33.2)

*Note*. CI = confidence interval; DID = difference‐in‐difference; IFA = iron and folic acid; MNCH = maternal, neonatal, and child health.

a Difference‐in‐difference impact estimate between baseline and endline adjusted for clustering effect at district and subdistrict levels.

### 3.5 Health workers' knowledge on maternal nutrition

Health workers in both areas had good knowledge of maternal nutrition (Table 3). Health workers' knowledge improved from baseline in both areas, but the improvements were significantly greater among health workers in nutrition‐focused MNCH areas for several indicators, including the importance of adequate nutrition of pregnant women, knowledge on why women should take IFA and calcium tablets, health risks for pregnant women if lacking iron in the diet, and beverages that decrease iron absorption.

**Table 3:** Health workers' knowledge on maternal nutrition, by study group and survey round

	Baseline		Endline		DID^a^ (95% CI)
	Nutrition‐focused MNCH	Standard MNCH	Nutrition‐focused MNCH	Standard MNCH	
	*n* = 105	*n* = 111	*n* = 111	*n* = 110	
	%	%	%	%	Percentage points
The importance of adequate nutrition for pregnant women					
For adequate weight gain of pregnant woman	71.4	69.4	92.8	69.1	21.5 (0.37, 43.4)
For child inside the womb to grow adequately/healthy	94.3	94.6	93.7	92.7	1.34 (−8.66, 11.3)
For a smart child with bright future	60.0	45.1	64.9	48.2	1.97 (−27.6, 31.6)
Extra costs due to doctors and medicine will be saved	24.8	19.8	36.9	17.3	14.8 (−19.3, 48.9)
Kind of food women should eat every day during pregnancy and postpartum period					
Fish/Meat	84.8	81.1	89.2	85.5	0.03 (−20.3, 20.4)
Egg	82.9	81.1	92.8	82.7	8.13 (−11.7, 27.9)
Milk/milk products	69.5	73.0	89.2	74.6	17.9 (−4.90, 40.7)
Dark green leafy vegetable	66.7	81.1	80.2	77.3	17.2 (−4.73, 39.2)
Yellow/orange vegetables/fruits	59.1	66.7	75.7	51.8	31.5 (4.33, 58.6)
Thick daal	39.1	39.6	61.3	52.7	8.51 (−14.5, 31.5)
The health risks for pregnant women of a lack of iron in the diet					
Develop anaemia/less iron in blood	81.0	80.2	89.2	80.0	8.46 (−10.0, 27.0)
Difficult delivery	53.3	45.1	69.4	49.1	12.1 (−19.5, 43.7)
Risk of dying during or after pregnancy	48.6	64.0	72.1	58.2	29.3 (−1.86, 60.6)
Some beverages decrease iron absorption when taken with meals					
Coffee	12.4	0.9	67.6	10.0	46.3 (28.4, 64.3)
Tea	20.0	8.1	86.5	20.9	53.6 (34.2, 72.9)
Reasons why pregnant women should take IFA tablets					
To reduce the risk of anaemia for pregnant women	81.9	82.9	89.2	88.2	1.74 (−19.7, 23.2)
To reduce the risk of low birthweight	41.9	35.1	56.8	38.2	12.1 (−26.9, 51.0)
To reduce the risk of excessive blood loss during/after delivery	34.3	37.8	68.5	30.9	41.1 (16.8, 65.5)
Reasons why PW should take calcium tablets					
To ensure adequate growth of child's bones and teeth	90.5	82.9	96.4	80.0	25.0 (0.51, 49.5)
To reduce the risk of pre‐eclampsia/eclampsia	33.3	27.0	71.8	23.6	41.7 (18.2, 65.2)

*Note*. CI = confidence interval; DID = difference‐in‐difference; IFA = iron and folic acid; MNCH = maternal, neonatal, and child health; PW = pregnant women.

a Difference‐in‐difference impact estimate between baseline and endline adjusted for clustering effect at district and subdistrict levels.

### 3.6 Coverage of service delivery

**Figure 2:**
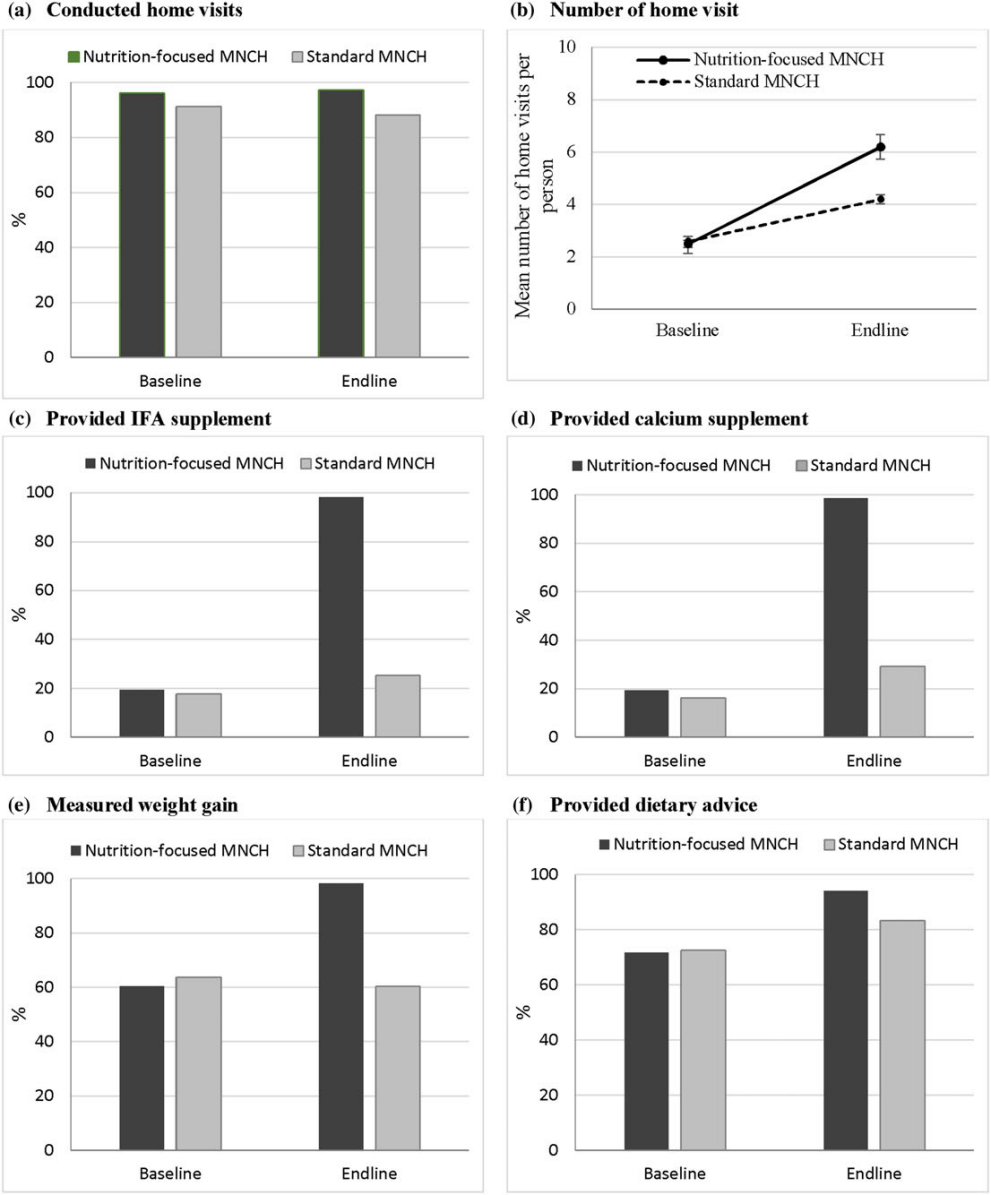
Coverage of nutrition service delivery, by study group and survey round. (a) Conducted home visits. (b) Number of home visits. (c) Provided iron and folic acid (IFA) supplement. (d) Provided calcium supplement. (e) Measured weight gain. (f) Provided dietary advice

The percentage of households visited by health workers was higher in nutrition‐focused MNCH than that in standard MNCH areas at endline (97% vs. 88%; Figure 2). Women in the nutrition‐focused MNCH areas were also visited at home more frequently during pregnancy (6.2 vs. 4.2 times). At endline, nearly all women in nutrition‐focused MNCH areas received free IFA and calcium tablets, which was much higher than those in standard MNCH areas (at 25% for IFA and 30% for calcium). Nearly all women in nutrition‐focused MNCH areas were weighed during their last pregnancy and also had weight measured earlier and more frequently compared with those in standard MNCH areas.

### 3.7 Quality of counselling service

During the household visits, a higher proportion of women in the nutrition‐focused MNCH areas received messages on maternal nutrition from health workers, including messages related to dietary quantity and quality, taking IFA and calcium daily, gaining weight, taking rest, and avoiding heavy workload during pregnancy (Table 4). In addition, a higher proportion of husbands in the nutrition‐focused MNCH areas received messages on how to support and ensure that their wives get adequate nutrition and enough rest during pregnancy and postpartum (results not shown).

**Table 4:** Quality of counselling services, by study group and survey round^a^

	Baseline		Endline		DID^a^ (95% CI)
	Nutrition‐focused MNCH	Standard MNCH	Nutrition‐focused MNCH	Standard MNCH	
	*n* = 1,000	*n* = 1,000	*n* = 1,000	*n* = 1,000	
	%	%	%	%	Percentage points
Messages on dietary quality					
Five types of food in addition to rice and thick dal	24.3	25.7	71.0	22.8	49.6 (34.1, 65.0)
Consume fish/meat daily	79.0	78.0	91.3	84.2	6.01 (−4.29, 16.5)
Consume egg daily	82.9	81.6	89.8	85.4	3.09 (−6.77, 12.9)
Consume milk/milk product daily	69.3	67.3	78.9	69.3	7.58 (−6.23, 21.4)
Consume dark green leafy vegetable daily	78.2	78.4	82.0	79.8	2.38 (−5.36, 10.1)
Consume yellow/orange fruit and vegetable daily	45.5	47.9	67.0	40.7	28.7 (10.8, 46.5)
Consume thick daal everyday	23.7	18.4	56.0	21.7	29.0 (11.6, 46.3)
Messages on dietary quantity					
A woman needs more energy and nutrients during pregnancy and lactation	67.2	71.7	82.8	66.6	20.7 (7.82, 33.5)
A variety of foods in additional amounts is required to meet the demands of the growing fetus	54.0	45.4	73.4	55.4	9.38 (−11.9, 30.7)
Messages received on taking IFA tablet					
IFA prevents anaemia	39.9	39.2	74.7	39.6	34.4 (18.9, 49.9)
IFA reduce risk of low‐birthweight baby	20.5	19.5	34.3	11.6	21.7 (4.05, 39.4)
IFA reduce risk of maternal death due to haemorrhage	11.5	11.3	28.9	9.1	19.6 (6.77, 32.5)
Messages received on taking calcium tablet					
Calcium helps baby/s bone and teeth development	25.4	26.1	70.0	33.7	37.0 (20.4, 53.5)
Calcium reduce risk of hypertension and eclampsia	10.5	6.7	52.7	8.4	40.5 (21.6, 59.3)
Messages received on taking rest while pregnant					
Take rest at least for 2 hr after lunch	85.3	77.7	90.8	81.0	2.19 (−6.09, 10.5)
Sleep for at least 8 hr at night	22.3	21.3	57.5	26.8	29.7 (12.1, 47.2)
Taking rest is important for the growth of the baby	16.1	16.2	28.6	20.0	8.73 (−3.97, 21.4)
Taking rest improves weight gain of the mother	8.1	6.7	16.0	7.5	7.11 (−4.06, 18.3)
Messages received on gaining weight during pregnancy					
A woman should gain 10–12‐kg weight during pregnancy	13.4	24.5	86.0	19.3	77.8 (65.4, 90.1)
Gaining weight indicates adequate growth of the fetus	27.1	30.5	44.3	32.5	15.2 (−5.89, 36.4)
Messages on avoid heavy workload	41.8	41.4	49.8	54.0	−4.57 (−27.5, 18.3)

*Note*. CI = confidence interval; DID = difference‐in‐difference; IFA = iron and folic acid; MNCH = maternal, neonatal, and child health.

a Difference‐in‐difference impact estimate between baseline and endline adjusted for clustering effect at district and subdistrict levels.

### 3.8 Perceptions about supervision

Overall perceptions about supervision were high in both groups at both baseline and endline (Table S1). A majority of the health workers felt that they received supportive feedback and enough guidance and support from their supervisors for their daily work. Health workers also reported that their supervisors often or always took their concerns into account when planning activities and brought them to the higher management level if needed, praised them when something was done well, and used times when mistakes were made as opportunities to help improve skills. No differences were observed between the nutrition‐focused MNCH and standard MNCH areas at both baseline and endline.

### 3.9 Incentives for health volunteers

At endline, about half of the health volunteers in the nutrition‐focused MNCH areas had ever received incentives from BRAC, and about one‐third received incentives in the last month for different activities, which was higher compared with those in the standard MNCH areas (at 10%). There was a positive association between incentives and coverage (β = 1.43 points) and quality of counselling service (β = 1.12 points; results not shown).

### 3.10 Effect of each implementation element on intake of IFA, calcium, and dietary diversity

**Figure 3:**
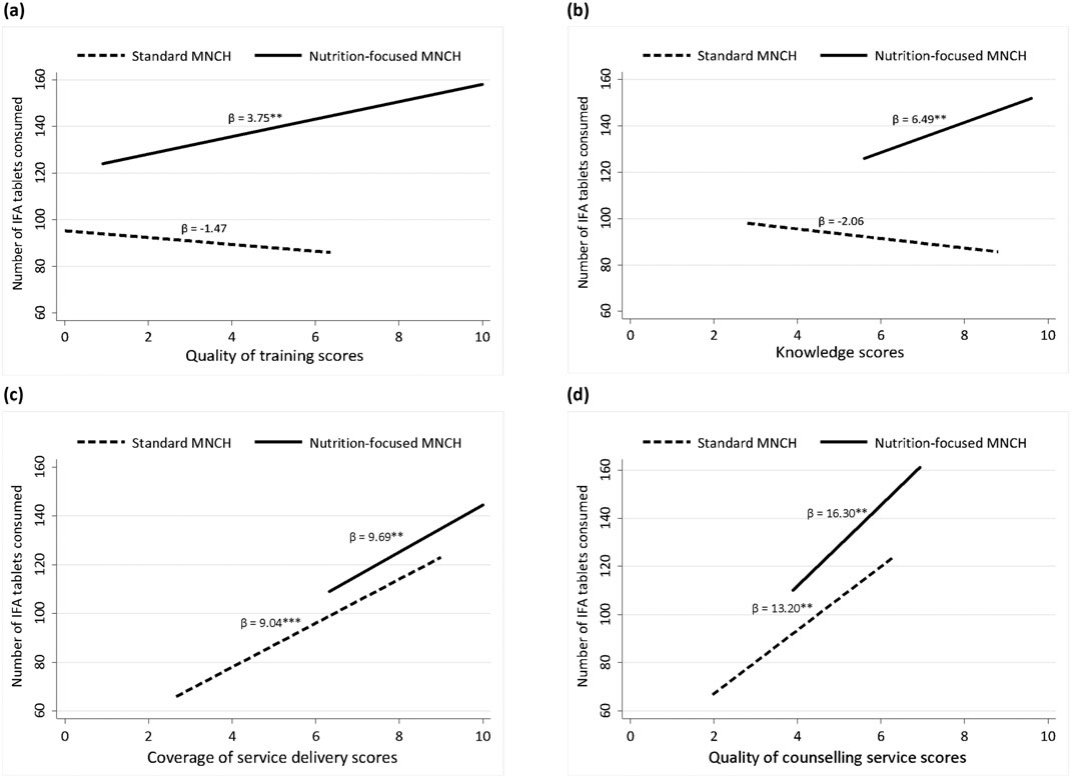
Regression models for consumption of iron and folic acid supplements for each domain of implementation. (a) Quality of training scores. (b) Knowledge scores. (c) Coverage of service delivery scores. (d) Quality of counselling service scores. *p < .05, **p < .01, ***p < .001. *p* for interaction = .027 for (a), .018 for (b), .871 for (c), and .474 for (d)

All villages in the nutrition‐focused MNCH areas showed a large shift towards higher training, knowledge, coverage, and quality of counselling service (Figure 3). Health workers' time commitment and their perceptions about supervision were high with no difference between study groups and, therefore, were not included in the path model. Incentives were only available for health volunteer data and, thus, were not included.

**Figure 4:**
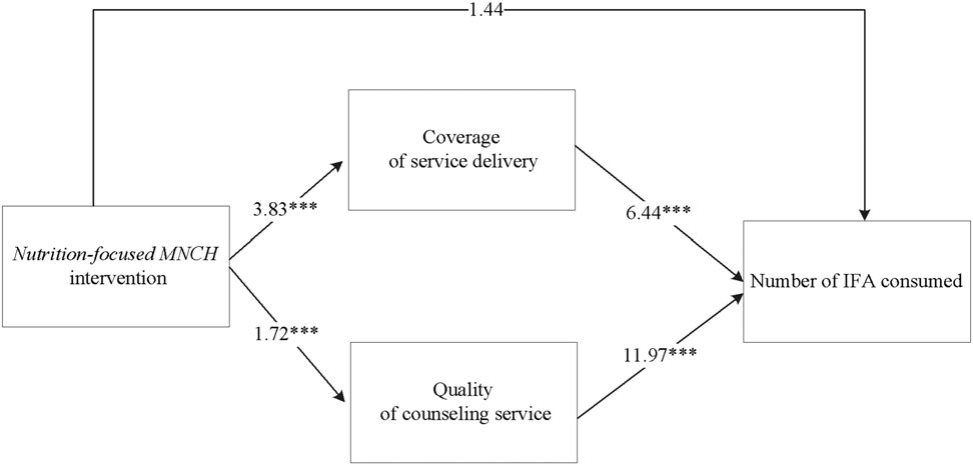
Path models for consumption of iron and folic acid supplements for coverage of service delivery and quality of counselling services (Values are coefficient; results are from separate path analytic models using health worker data; indirect effects through coverage of service delivery: 52.9% of total effect; through quality of counselling service: 44.1% of total effect). *p < .05, **p < .01, ***p < .001

For IFA consumption, intervention area interacted with the training and knowledge elements; in nutrition‐focused MNCH areas, villages with better training and knowledge scores had higher IFA consumption (β = 3.75 and 6.49, respectively); in contrast, no association was observed in standard MNCH areas (Figure 3). Path analysis was conducted for coverage of service delivery and counselling quality because there was no interaction between the intervention area and these elements. The indirect paths, estimated as the products of the regression coefficients for each path, for coverage of service delivery and quality of counselling service explained 52.9% and 44.1%, respectively, of the difference in IFA supplement consumption (Figure 4). Similar results were found for calcium supplement consumption.

For dietary diversity, intervention area interacted with coverage of service delivery, such that no association was seen between coverage score and number of food groups consumed in the standard MNCH areas, but a one‐point difference in coverage score was associated with a 0.54 higher number of food groups consumed in the nutrition‐focused MNCH areas. Variation among villages in dietary diversity was largely explained by variation in quality of counselling services (60%), with less explained by training (7%) and knowledge (11%; results not shown).

## 4 DISCUSSION

This paper examined the elements of the implementation process through which the Alive & Thrive nutrition‐focused MNCH intervention intended to improve maternal nutrition practices and revealed that the integration of the nutrition‐focused intervention into the MNCH programme was feasible and well‐implemented. Training quality, health workers' knowledge, coverage of service delivery, and quality of counselling service increased significantly over time and were higher at endline in the nutrition‐focused MNCH areas, compared with those in the standard MNCH areas. Furthermore, the differences in coverage of service delivery and counselling quality explained a large portion of the variation among villages for intake of IFA and calcium supplements, and differences in quality of counselling services explained 60% of the programme's impact on women's dietary diversity during pregnancy.

The intervention implementation was successful for several reasons. The intervention was implemented by BRAC, a large non‐governmental organization with more than 40 years of experience in implementing community‐based health interventions throughout Bangladesh. In particular, BRAC has been providing community‐based MNCH interventions as part of prenatal healthcare since 2008 (Afsana et al., 2009; Rahman et al., 2015); thus; management was in place to facilitate operations, with working structures to carry out monitoring and supportive supervision. BRAC's cadres of health workers and volunteers are highly motivated and well‐supervised, allowing reach to all the pregnant and lactating women and young children with the integrated intervention with little additional resources and support structures. These FLWs live in the communities and are trusted by community members, and their services are well received by those whom they serve (Rahman, Leppard, Rashid, Jahan, & Nasreen, 2016). The intervention was also carefully designed with questions of local relevance and delivery feasibility in mind, and using evidence‐based findings to address barriers to recommended practices, improve FLW training modules, target home visits and counselling, and standardize messages (Alam et al., 2015).

Training is an important element to developing and maintaining health worker competencies for delivering quality services (World Health Organization, 2015). In our study, we observed an association between higher training quality or knowledge scores and higher consumption of IFA and calcium supplements in nutrition‐focused MNCH areas, but no association was observed in standard MNCH areas. It is possible that the more intensive and in‐depth training contents with interactive training methods in nutrition‐focused MNCH areas contributed to these differences. Going beyond general training, in nutrition‐focused MNCH areas, trainings have focused on clear and relevant instructions on counselling approach for FLWs, together with budget discussion on practical and feasible family foods, as well as counting and recording micronutrient tablets. All these careful trainings on micro level task has helped FLWs' success in delivering counselling services to mothers and family members and likely helped achieve high consumption among women.

In addition to improving FLWs' knowledge and skills through training, multiple strategies were applied in nutrition‐focused MNCH areas to improve the performance of FLWs in delivering nutrition interventions as part of their MNCH activities, including clearly defined roles and responsibilities, allocation of specific nutrition tasks across categories of FLWs and staff, intensified and frequent training and orientation sessions, simplified job aids and tools for service delivery, frequent nutrition‐focused supervision, continuous refresher training, timely feedback using monitoring data, performance‐based cash incentives, and assured micronutrient supplies and scales. These differences likely led to the significant improvements in coverage and quality of service delivery which in turn contributed to the observed impacts on IFA and calcium supplement consumption.

In relation to the outcomes, intake of micronutrient supplements and food intake (dietary diversity) are quite different behaviours. For consumption of IFA and calcium supplements, coverage and quality were important in both areas; thus, increased service contacts and counselling quality appeared to have influenced women to continue taking the micronutrient supplements for longer throughout of their pregnancy. For dietary diversity, quality of counselling service explained the most variation among villages in both areas, but coverage was only associated with dietary diversity in the nutrition‐focused MNCH areas. Unlike taking IFA and calcium tablets, which were distributed for free to the women in the nutrition‐focused MNCH areas, dietary diversity is a more complex behaviour influenced by sociocultural and economic constraints to acquisition of diverse foods, particularly in the low‐income settings of our study. For instance, during formative research, poverty emerged as the main barrier to following the dietary recommendations, and women gave low priority to their own diets as compared with those of other family members, an issue typical in the South Asian culture (Schuler, 2015). Providing adequate advice and support to address these constraints and promote consumption of varied diets requires not only good counselling skills but also clear instruction and practical discussion with family members on locally available and affordable foods within family‐limited budget, which was the focus of the intervention in nutrition‐focused MNCH areas and is likely the reason we observed the significant associations with both counselling quality and service exposure in this area.

Although FLWs' knowledge of key maternal nutrition issues improved over time in intervention areas, there are some topics, such as the importance of proper nutrition for pregnant women and reasons why pregnant women should take IFA and calcium supplements, that still need particular attention for further improvement. Coverage is high, but counselling quality can improve further. For example, although many FLWs delivered general messages on diet diversity and IFA and calcium consumption, specific messages in each theme were not delivered, particularly messages on consumption of thick dal, nutritious snacks, or extra foods and messages on rest and weight gain during pregnancy. Understanding why certain nutrition messages are delivered, not delivered, or even modified during interpersonal counselling will help strengthen the implementation. It is plausible that FLWs recall or choose to deliver certain messages based on their own experiences, beliefs and values, communication skills, perceived constraints, and/or reactions from beneficiaries. Further qualitative study will be useful to explore the factors affecting the delivery of behaviour change messages intended to address maternal undernutrition.

A limitation of this paper was the lack of direct observations of FLWs' activities, thus limiting our full understanding of the quality of service delivery during home visits. Also, perceptions of supervision were high overall, which may be due to courtesy bias where employees tend to avoid expressing negative opinions about their supervisors, resulting in overly favourable responses. Because this study was carried out in the context of a well‐functioning and robust MNCH platform, the generalizability of intervention effects should be interpreted with caution in other contexts, particularly with weaker health systems.

Our study has several strengths. The rich data at both FLW and household levels enabled a theory‐driven approach to study implementation across multiple elements. By analysing the differences between the nutrition‐focused MNCH and standard MNCH areas within the context of a cluster‐randomized programme evaluation, this paper strengthens the plausibility that improvements in various implementation elements are attributable to the intervention. Additionally, the path analysis allows us to quantify the contribution of different implementation elements in explaining intervention impacts. Finally, the study contributes to the literature on implementation science in nutrition, providing insights on where and how maternal nutrition interventions can be improved. Lesson learned from this intervention, together with training manuals, job aids, and other materials, are being shared with the Bangladesh government and elsewhere in the efforts to integrate and reinforce nutrition interventions delivered through routine healthcare services (Billah et al., 2017).

## 5 CONCLUSIONS

The integration of the maternal nutrition‐focused intervention into an existing MNCH programme was feasible and well‐implemented. Although differences in coverage and counselling quality most explained impacts, all intervention elements—particularly FLW training and performance—were likely important to achieving impact.

## CONFLICTS OF INTEREST

The authors declare that they have no conflicts of interest.

## CONTRIBUTIONS

PHN contributed in the study design, coordinating data collection, developing research questions and conducting the statistical analysis of data, drafting and revising the manuscript. EAF contributed to the study design, provided guidance to the statistical analysis and inputs for the manuscript, and critically reviewed and revised the manuscript. TS contributed in developing research questions and interpreting results and provided inputs to the sections on intervention design and implementation. SSK reviewed and provided inputs for data interpretation and participated in drafting and revising the manuscript. SA contributed in developing research questions, data interpretation, and comments for the manuscript. LMT conducted the statistical analysis of data and prepared tables and figures for the manuscript. ZM and BA provided inputs to the sections on intervention design and implementation and provided comments on the manuscript. PM contributed in the study design, contributed in developing research questions, and critically reviewed and revised the manuscript. All authors read and approved the final submitted manuscript.

## Supporting information

Table S1: Health workers' supervision experience, by study group and survey round^1^

## References

[mcn12613-bib-0001] Afsana K. , Alam A. , Chowdhury M. , Rhode J. , Ahmed F. , Rahman H. …, Kairy SN. (2009). MANOSHI: A programme for improving maternal, neonatal and child health in the urban slums of Bangladesh. MANOSHI Working Paper Series No. 1. ICDDR,B, BRAC. Bangladesh.

[mcn12613-bib-0002] Alam, A. , Rasheed, S. , Khan, N. U. , Sharmin, T. , Huda, T. M. , Arifeen, S. E. , & Dibley, M. J. (2015). How can formative research inform the design of an iron‐folic acid supplementation intervention starting in first trimester of pregnancy in Bangladesh? BMC Public Health, 15, 374. 25887449 10.1186/s12889-015-1697-2 PMC4425912

[mcn12613-bib-0003] Arsenault, J. E. , Yakes, E. A. , Islam, M. M. , Hossain, M. B. , Ahmed, T. , Hotz, C. , … Brown, K. H. (2013). Very low adequacy of micronutrient intakes by young children and women in rural Bangladesh is primarily explained by low food intake and limited diversity. The Journal of Nutrition, 143, 197–203. 23256144 10.3945/jn.112.169524

[mcn12613-bib-0004] Avula, R. , Menon, P. , Saha, K. K. , Bhuiyan, M. I. , Chowdhury, A. S. , Siraj, S. , … Frongillo, E. A. (2013). A program impact pathway analysis identifies critical steps in the implementation and utilization of a behavior change communication intervention promoting infant and child feeding practices in Bangladesh. The Journal of Nutrition, 143, 2029–2037. 24068790 10.3945/jn.113.179085

[mcn12613-bib-0005] Billah, S. M. , Saha, K. K. , Khan, A. N. S. , Chowdhury, A. H. , Garnett, S. P. , Arifeen, S. E. , & Menon, P. (2017). Quality of nutrition services in primary health care facilities: Implications for integrating nutrition into the health system in Bangladesh. PLoS One, 12, e0178121. 28542530 10.1371/journal.pone.0178121 PMC5436890

[mcn12613-bib-0006] Campbell, M. , Fitzpatrick, R. , Haines, A. , Kinmonth, A. L. , Sandercock, P. , Spiegelhalter, D. , & Tyrer, P. (2000). Framework for design and evaluation of complex interventions to improve health. BMJ, 321, 694–696. 10987780 10.1136/bmj.321.7262.694 PMC1118564

[mcn12613-bib-0007] Carroll, C. , Patterson, M. , Wood, S. , Booth, A. , Rick, J. , & Balain, S. (2007). A conceptual framework for implementation fidelity. Implementation Science, 2, 40. 18053122 10.1186/1748-5908-2-40 PMC2213686

[mcn12613-bib-0008] Damschroder, L. J. , Aron, D. C. , Keith, R. E. , Kirsh, S. R. , Alexander, J. A. , & Lowery, J. C. (2009). Fostering implementation of health services research findings into practice: A consolidated framework for advancing implementation science. Implementation Science, 4, 50. 19664226 10.1186/1748-5908-4-50 PMC2736161

[mcn12613-bib-0009] Duerden, M. D. , & Witt, P. A. (2012). Assessing program implementation: What it is, why it's important, and how to do it. Journal of Extension, 50, 1–8.

[mcn12613-bib-0010] Durlak, J. A. , & Dupre, E. P. (2008). Implementation matters: a review of research on the influence of implementation on program outcomes and the factors affecting implementation. American Journal of Community Psychology, 41, 327–350. 18322790 10.1007/s10464-008-9165-0

[mcn12613-bib-0011] Fao & Fhi360 (2016). Minimum dietary diversity for women: A guide for measurement. Rome: FAO.

[mcn12613-bib-0012] Fixsen D. J. , Naoom S. F. , Blase K. A. , Friedman R. F. , Wallace F. & Wallace P. (2005). Implementation research: A synthesis of the literature. Tampa, FL: University of South Florida, Louis de la Parte Florida Mental Health Institute, The National Implementation Research Network (FMHI Publication #231).

[mcn12613-bib-0013] Gertler, P. , Martinez, S. , Premand, P. , Rawlings, L. , & Vermeersch, C. (2011). Impact evaluation in practice. Washington, DC: World Bank Publications.

[mcn12613-bib-0014] Hayes, R. J. , & Moulton, L. H. (2009). Cluster randomized trials. Boca Raton: Chapman & Hall/CRC Press.

[mcn12613-bib-0015] Hyder, S. M. , Persson, L. A. , Chowdhury, M. , Lonnerdal, B. O. , & Ekstrom, E. C. (2004). Anaemia and iron deficiency during pregnancy in rural Bangladesh. Public Health Nutrition, 7, 1065–1070. 15548345 10.1079/PHN2004645

[mcn12613-bib-0016] Kim, S. S. , Ali, D. , Kennedy, A. , Tesfaye, R. , Tadesse, A. W. , Abrha, T. H. , … Menon, P. (2015). Assessing implementation fidelity of a community‐based infant and young child feeding intervention in Ethiopia identifies delivery challenges that limit reach to communities: A mixed‐method process evaluation study. BMC Public Health, 15, 316. 25879417 10.1186/s12889-015-1650-4 PMC4392481

[mcn12613-bib-0017] Kline, R. (2011). Principles and practice of structural equation modeling (Third ed.). New York, NY: Guilford Press.

[mcn12613-bib-0018] Leroy, J. L. , & Menon, P. (2008). From efficacy to public health impact: a call for research on program delivery and utilization in nutrition. The Journal of Nutrition, 138, 628–629. 18287377 10.1093/jn/138.3.628

[mcn12613-bib-0019] Loechl, C. U. , Menon, P. , Arimond, M. , Ruel, M. T. , Pelto, G. , Habicht, J. P. , & Michaud, L. (2009). Using programme theory to assess the feasibility of delivering micronutrient Sprinkles through a food‐assisted maternal and child health and nutrition programme in rural Haiti. Maternal & Child Nutrition, 5, 33–48. 19161543 10.1111/j.1740-8709.2008.00154.x PMC6860826

[mcn12613-bib-0020] Menon, P. , Mbuya, M. , Habicht, J. P. , Pelto, G. , Loechl, C. U. , & Ruel, M. T. (2008). Assessing supervisory and motivational factors in the context of a program evaluation in rural Haiti. The Journal of Nutrition, 138, 634–637. 18287379 10.1093/jn/138.3.634

[mcn12613-bib-0021] Nguyen, P. H. , Kim, S. S. , Sanghvi, T. , Mahmud, Z. , Tran, L. M. , Shabnam, S. , … Menon, P. (2017). Integrating nutrition interventions into an existing maternal, neonatal, and child health program increased maternal dietary diversity, micronutrient intake, and exclusive breastfeeding practices in Bangladesh: Results of a cluster‐randomized program evaluation. Journal of Nutrition(in press, 147, 2326–2337. 29021370 10.3945/jn.117.257303 PMC5697969

[mcn12613-bib-0022] Nguyen, P. H. , Menon, P. , Keithly, S. C. , Kim, S. S. , Hajeebhoy, N. , Tran, L. M. , … Rawat, R. (2014). Program impact pathway analysis of a social franchise model shows potential to improve infant and young child feeding practices in Vietnam. The Journal of Nutrition, 144, 1627–1636. 25143372 10.3945/jn.114.194464

[mcn12613-bib-0023] NIPORT . (2011). National Institute of Population Research and Training (NIPORT) , Mitra and Associates, and ICF International. Bangladesh Demographic and Health Survey 2011. Dhaka, Bangladesh, and Rockville, Maryland, USA.

[mcn12613-bib-0024] Rahman, A. , Leppard, M. , Rashid, S. , Jahan, N. , & Nasreen, H. E. (2016). Community perceptions of behaviour change communication interventions of the maternal neonatal and child health programme in rural Bangladesh: an exploratory study. BMC Health Services Research, 16, 389. 27530405 10.1186/s12913-016-1632-y PMC4987986

[mcn12613-bib-0025] Rahman, M. , Jhohura, F. T. , Mistry, S. K. , Chowdhury, T. R. , Ishaque, T. , Shah, R. , & Afsana, K. (2015). Assessing community based improved maternal neonatal child survival (IMNCS) program in rural Bangladesh. PLoS One, 10, e0136898. 26340672 10.1371/journal.pone.0136898 PMC4560389

[mcn12613-bib-0026] Rawat, R. , Nguyen, P. H. , Ali, D. , Saha, K. , Alayon, S. , Kim, S. S. , … Menon, P. (2013). Learning how programs achieve their impact: Embedding theory‐driven process evaluation and other program learning mechanisms in Alive & Thrive. Food and Nutrition Bulletin, 34, S212–S225. 24261078 10.1177/15648265130343S207

[mcn12613-bib-0027] Robert, R. C. , Gittelsohn, J. , Creed‐Kanashiro, H. M. , Penny, M. E. , Caulfield, L. E. , Narro, M. R. , & Black, R. E. (2006). Process evaluation determines the pathway of success for a health center‐delivered, nutrition education intervention for infants in Trujillo, Peru. The Journal of Nutrition, 136, 634–641. 16484536 10.1093/jn/136.3.634

[mcn12613-bib-0028] Robert, R. C. , Gittelsohn, J. , Creed‐Kanashiro, H. M. , Penny, M. E. , Caulfield, L. E. , Narro, M. R. , … Black, R. E. (2007). Implementation examined in a health center‐delivered, educational intervention that improved infant growth in Trujillo, Peru: Successes and challenges. Health Education Research, 22, 318–331. 16945983 10.1093/her/cyl078

[mcn12613-bib-0029] Saunders, R. P. , Evans, M. H. , & Joshi, P. (2005). Developing a process‐evaluation plan for assessing health promotion program implementation: A how‐to guide. Health Promotion Practice, 6, 134–147. 15855283 10.1177/1524839904273387

[mcn12613-bib-0030] Schuler S. (2015). Alive &Thrive formative research on maternal nutrition in Bangladesh (unpublished).

[mcn12613-bib-0031] Shekar, M. (2008). Delivery sciences in nutrition. Lancet, 371, 1751. 10.1016/S0140-6736(08)60757-6 18502295

[mcn12613-bib-0032] Stoltzfus, R. J. (2008). Research needed to strengthen science and programs for the control of iron deficiency and its consequences in young children. The Journal of Nutrition, 138, 2542–2546. 19022987 10.3945/jn.108.094888

[mcn12613-bib-0033] Vanderweele, T. (2015). Explanation in causal inference. Methods for mediation and interaction. New York: Oxford University Press.

[mcn12613-bib-0034] WHO . (2015). Strengthening the capacity of community health workers to deliver care for sexual, reproductive, maternal, newborn, child and adolescent health. http://www.who.int/workforcealliance/knowledge/resources/who_2015_h4_chws_srmncah.pdf?ua=1. Geneva, Switzerland: World Health Organization.

