# Supplementary material for: Importance of coverage and quality for impact of nutrition interventions delivered through an existing health programme in Bangladesh
Source: Matern Child Nutr. 2018 Apr 14;14(4):e12613. doi: 10.1111/mcn.12613 (PMC6175250; doi:10.1111/mcn.12613)
Supplement: Supplementary file 1 — Table S1: Health workers' supervision experience, by study group and survey round1 [file MCN-14-e12613-s001.docx]

**Supplemental Table 1: Health workers’ supervision experience, by study group and survey round^1^**

|  | **Baseline** | | **Endline** | |
| --- | --- | --- | --- | --- |
|  | **Nutrition-focused MNCH** | **Standard**  **MNCH** | **Nutrition-focused MNCH** | **Standard**  **MNCH** |
|  | **n= 105** | **n= 111** | **n= 111** | **n= 110** |
|  | **Mean ± SD** | **Mean ± SD** | **Mean ± SD** | **Mean ± SD** |
| My supervisor keeps me informed about the follow-up of my concerns/worries | 4.5 ± 0.7 | 4.5 ± 0.7 | 4.4 ± 0.7 | 4.4 ± 0.7 |
| My supervisor informs me about upcoming trainings/meetings, etc., in a timely fashion | 4.7 ± 0.6 | 4.8 ± 0.4 | 4.8 ± 0.5 | 4.7 ± 0.5 |
| My supervisor respects my fixed monthly activities when planning other meetings | 4.7 ± 0.6 | 4.5 ± 0.8 | 4.4 ± 0.8 | 4.5 ± 0.7 |
| My supervisor consults with me before making changes to the activities that I am involved in. | 4.5 ± 0.7 | 4.3 ± 0.9 | 4.4 ± 0.8 | 4.4 ± 0.7 |
| When I make a mistake on the job, my supervisor scolds me | 2.6 ± 1.3 | 2.2 ± 1.2 | 1.7 ± 1.2 | 1.7 ± 1.0 |
| My supervisor praises me when I do something really well | 4.2 ± 1.0 | 3.9 ± 1.1 | 4.1 ± 1.0 | 4.1 ± 1.0 |
| My supervisor helps me to organize my time and activities in an efficient manner | 4.6 ± 0.7 | 4.3 ± 0.9 | 4.4 ± 0.7 | 4.3 ± 0.8 |
| My supervisor ensures that I have enough of the supplies that I need to do my daily work. | 4.6 ± 0.5 | 4.5 ± 0.6 | 4.5 ± 0.5 | 4.5 ± 0.5 |
| When I disagree with my supervisor I feel safe to express my opinion | 3.9 ± 1.0 | 4.1 ± 0.9 | 4.2 ± 0.9 | 4.1 ± 0.9 |
| The way the supervisor provides feedback on my performance at the CHC inspires me to be my best | 4.4 ± 0.6 | 4.3 ± 0.7 | 4.1 ± 0.6 | 4.3 ± 0.7 |
| My supervisor takes into account/considers my suggestions to improve things | 4.5 ± 0.7 | 4.4 ± 0.7 | 4.3 ± 0.7 | 4.4 ± 0.5 |
| My supervisor works with me to identify solutions to program activity related problems | 4.5 ± 0.5 | 4.4 ± 0.6 | 4.4 ± 0.6 | 4.5 ± 0.6 |
| I feel that my supervisor is sympathetic to my problems/cares about my problems | 4.4 ± 0.7 | 4.3 ± 0.7 | 4.3 ± 0.6 | 4.4 ± 0.7 |
| My supervisor gives me enough guidance and structure to help me do my job | 4.5 ± 0.5 | 4.5 ± 0.5 | 4.5 ± 0.5 | 4.5 ± 0.6 |
| My supervisor uses times when I make mistakes or don’t perform well as opportunities to help me improve my skills | 4.5 ± 0.6 | 4.5 ± 0.6 | 4.4 ± 0.6 | 4.4 ± 0.6 |
| **Total supervision score (1-10)** | **8.7** ± **0.7** | **8.4** ± **0.8** | **8.2** ± **0.7** | **8.0** ± **0.9** |

^1^Differences in groups at endline were tested using ordinary least squares regression models, adjusting for clustering effect at district and sub-district levels.
